# Prevalence of abnormally invasive placenta among deliveries in mainland China: A PRISMA-compliant Systematic Review and Meta-analysis

**DOI:** 10.1097/MD.0000000000006636

**Published:** 2017-04-21

**Authors:** Dazhi Fan, Song Li, Shuzhen Wu, Wen Wang, Shaoxin Ye, Qing Xia, Li Liu, Jinping Feng, Song Wu, Xiaoling Guo, Zhengping Liu

**Affiliations:** a Foshan Institute of Fetal Medicine; b Department of Obstetrics, Southern Medical University Affiliated Maternal & Child Health Hospital of Foshan, Foshan, Guangdong; c Department of Epidemiology and Biostatistics, School of Public Health, Anhui Medical University, Hefei; d Chaohu Hospital Affiliated Anhui Medical University, Chaohu, Anhui; e Department of Library, the First Affiliated Hospital, College of Medicine, Zhejiang University, Hangzhou, Zhejiang; f School of Integrated Traditional and Western Medicine, Anhui University of Chinese Medicine, Hefei, Anhui, China.

**Keywords:** abnormally invasive placenta, mainland China, meta-analysis, prevalence

## Abstract

**Background:**

Abnormally invasive placenta (AIP) or placenta accreta (PA) is considered numerous adverse maternal and fetal-neonatal complications. There has been no detailed study to characterize the prevalence of AIP among deliveries in mainland China.

**Objectives:**

We aimed to fill this gap and obtained overall and regional estimates of AIP prevalence via a systematic review and meta-analysis.

**Methods:**

This systematic review and meta-analysis was totally performed following the Meta-analysis of Observational Studies in Epidemiology guidelines for systematic reviews of observational studies, and the Preferred Reporting Items for Systematic reviews and Meta-Analysis statement for reporting systematic reviews and meta-analysis.

**Results:**

A total of 23 articles (including 350,939) were included. Most studies (22) focused on the placenta increta (PI), only 2 articles in PA, and none in placenta percreta (PP). In this meta-analysis, the overall prevalence of AIP was 0.22% (95% confidence interval 0.18%–0.27%) in a heterogeneous set of studies (*I*^2^ = 93.5%). In a subgroup, the prevalence of PA and PI was 0.48% and 0.23%, respectively. Stratified analyses of PI found that the prevalence was similar in North (0.23% [0.14%–0.32%]) and South (0.23% [0.15%–0.32%]), and lower in Central (0.20% [0.09–0.31%]); the inlanders (0.17% (0.12%–0.23%]) had a lower prevalence of PI than those living in coastal areas (0.24% [0.35%–0.63%]). As time goes on, the prevalence, from 0.03% (0.02%–0.04%) in 1970 to 1979 to 0.48% (0.30%–0.66%) in 2010 to present, was higher and higher. The different prevalence was also found in different in maternal age groups.

**Conclusions:**

This first systematic review and meta-analysis found that the prevalence differed among different geographic areas and maternal age groups. The results would be useful for the design of abnormally invasive placenta planning and implementation adequate health care systems and treatment programs in mainland China.

## Abstract 2

Supplemental Digital Content is available in the text

## 1 Introduction

Abnormally invasive placenta (AIP) or placenta accreta (PA), was defined as trophoblastic attachment to the myometrium without intervening decidua, and referred to the entire spectrum of conditions including PA, placenta increta (PI), and placenta percreta (PP).^[1]^ The trophoblast was attached to the myometrium in PA, if the trophoblast actually invaded into the myometrium, it was termed PI, and if it invaded through the myometrium beyond the serosa and into surrounding structures such as the bladder or intestines, it was termed PP.^[2]^ It was considered numerous adverse maternal and fetal-neonatal complications. The primary maternal complication was life-threatening peripartum hemorrhage, which can lead to hysterectomy, disseminated intravascular coagulation, multisystem organ failure, acute respiratory distress syndrome, and even death.^[3,4]^ It was also associated with an increased risk of preterm birth. The average gestational age of delivery of accreta was typically 34 to 35 weeks of gestation,^[5,6]^ typically as a result of medically indicated preterm birth and associated complications of prematurity.

PA was associated strongly with the combination of previous cesarean section and placenta previa.^[7,8]^ Other risk factors included increasing maternal age, multiparity, smoking, in vitro fertilization, and a short interval between a previous cesarean delivery and subsequent pregnancy.^[9–12]^ The prevalence of PA has steadily increased over the past several decades.^[13]^ In the United States, it has currently increased to about 1 in 500 pregnancies.^[14]^ A recent study noted that the prevalence of PA was approximately 0.50% in mainland China,^[15]^ nearly 2.5 times higher than the level reported in the United States. The World Health Organization (WHO) Global Survey reported that the rate of cesarean delivery was as high as 46.2% in China,^[16]^ whereas in some cases, it was up to 80%,^[17]^ which was now higher than the upper limit of 15% recommended by the WHO's guidelines.^[18]^ Through a Preferred Reporting Items for Systematic Reviews and Meta-Analysis (PRISMA)-compliant systematic review and meta-analysis, we recently found that placenta previa, the most important risk factor for PA, was a high-burden disease in Mainland China.^[19]^ Meanwhile, the Chinese government instituted a policy in 2015 to allow 2 children in each family.^[20]^ Not surprisingly, with this relaxation of “one-child policy,” the incidence of advanced maternal age will rise further.

Owing to several factors such as the increasing rates of cesarean section, increasing rates of advanced maternal age, and ongoing nutritional transition, the prevalence of PA will rise in recent years in mainland China. Because of the time-consuming and high cost of epidemiological survey in the largest population in the world, a national epidemiological survey of PA, including most of provinces, has never been performed to date in the pregnancies population in a vast territory of mainland China. Although previous studies provided lots of valuable information, they focused on one or several provinces rather than nationally representative sample of pregnancies population. The data of PA remain incompletely limited. There has been no systematic review and meta-analysis to characterize the prevalence of PA among deliveries in mainland China.

Therefore, we aim to fill this gap and will answer the following questions: what is the prevalence of AIP (PA, PI, or/and PP) among deliveries in mainland China? Which factors are associated with PA? And, what is the comprehensive picture of PA in different geographical region in mainland China? The result would be useful for the design of PA planning and implementation adequate health care systems and treatment programs in mainland China.

## 2 Methods

This systematic review protocol has been published in the PROSPERO International Prospective Register of systematic reviews (http://www.crd.york.ac.uk/PROSPERO/), and the registration number is CRD42016035833. As our previous studies,^[21,22]^ the present research was also totally performed a systematic review, following the Meta-analysis of Observational Studies in Epidemiology (MOOSE) guidelines for systematic reviews of observational studies,^[23]^ and the PRISMA statement for reporting systematic reviews and meta-analysis (Supplementary Table 1).^[24]^ Being a systematic review, no ethical approval was needed for this manuscript.

### 2.1 Search strategy and selection criteria

A literature search of PubMed, Cochrane Library, Elsevier Science Direct, the Chinese Biological Medical Literature database (CBM), the Chinese National Knowledge Infrastructure database (CNKI), Chinese WanFang Database, and Chinese VIP database were conducted using the following subject terms “abnormally invasive placenta,” “placenta accreta,” “placenta increta,” “placenta percreta,” “adherent placenta,” “China,” “Chinese.” Relevant eligible literatures were also scanned through cross-references of identified in the reference lists within both original and review articles. The search was limited to include only references in English and Chinese language, and the search time was updated in December 2016.

The studies were included in our analysis if they meet the following criteria: studies either provided the number of cases of AIP (PA, PI, or/and PP) and the total number of deliveries or births or sufficient data for calculating the prevalence in the population; studies were conducted in mainland China and because of cultural differences from mainland China, studies from Taiwan, Hong Kong, and Macao were excluded^[19,25]^; articles were published in English or Chinese. The studies were excluded based on the following criteria: case series with small sample size (<50 participants), letters, reviews and editorials; the full data were not accessible even after request from the corresponding author. If multiple publications covered the same study, the most comprehensive one reporting the largest sample size was considered.

### 2.2 Data extraction

After initial evaluation, 2 reviewers (DF and SW) independently and carefully evaluated the articles and performed the data extraction according to the selection criteria. We extracted the following variables: first author, years published, survey years, study location (including cities and provinces), age (mean ± standard deviation or median, range), the number of cases of AIP (PA, PI, or/and PA), and the total sample size. When discrepancies existed, discussion was performed or via consultation with another reviewer (WW) until a consensus was reached. If necessary, the corresponding author of the published studies was contacted to provide relevant information for our analysis.

### 2.3 Methodological quality assessment

The methodological quality of each study was independently assessed by 2 reviewers (DF and QX) via the Reporting of Observational Studies in Epidemiology (STROBE) guideline.^[26]^ STROBE was a 5-item instrument with a low-risk (score = 2), moderate-risk (score = 1), and-high risk (score = 0) response option, and the total score, ranged from 0 to 10, represented the summary assessment of bias risk for each study. When there was a disagreement, it was solved by consensus of the whole team.

### 2.4 Statistical analysis

Individual and pooled prevalence and 95% confidence interval (95% CI) were calculated for each of all the included studies using the STATA 12.0 (Stata Corp, College Station, TX). Before performed an inverse-variance weighted, prevalence was transformed via the Freeman-Tukey double arcsine method.^[27]^ Statistical heterogeneity was evaluated by the *χ*^2^ test on Q statistic, which was quantified by the *I*^2^ values, assuming that *I*^2^ values 25%, 50%, and 75% were nominally assigned as low, moderate, and high estimates, respectively.^[28]^ The inverse variance methods and DerSimonian-Laird random-effects model meta-analysis was used to determine the weight of each study.^[29]^ To investigate potential sources of heterogeneity, subgroup analyses and meta-regression were performed to find any possible sources using the following grouping variables: geographical region and location, maternal age, data collection period, study quality, and hospital level. Geographical area was divided according to the commonly used zoning map of China^[30,31]^; study quality on the basis of the total score of STROBE result; and hospital classification based on the Chinese hospital grade query system.^[19,32]^ Sensitivity analysis was performed to assess whether ≥1 studies influenced the overall results. A funnel plot (prevalence versus standard error) and the method of Begg and Egger test were used to explore the potential publication bias.^[33]^ And *P* ≤ +.05 will be indicated the presence of statistically significant.

## 3 Results

### 3.1 Search results and characteristics

**Figure 1:**
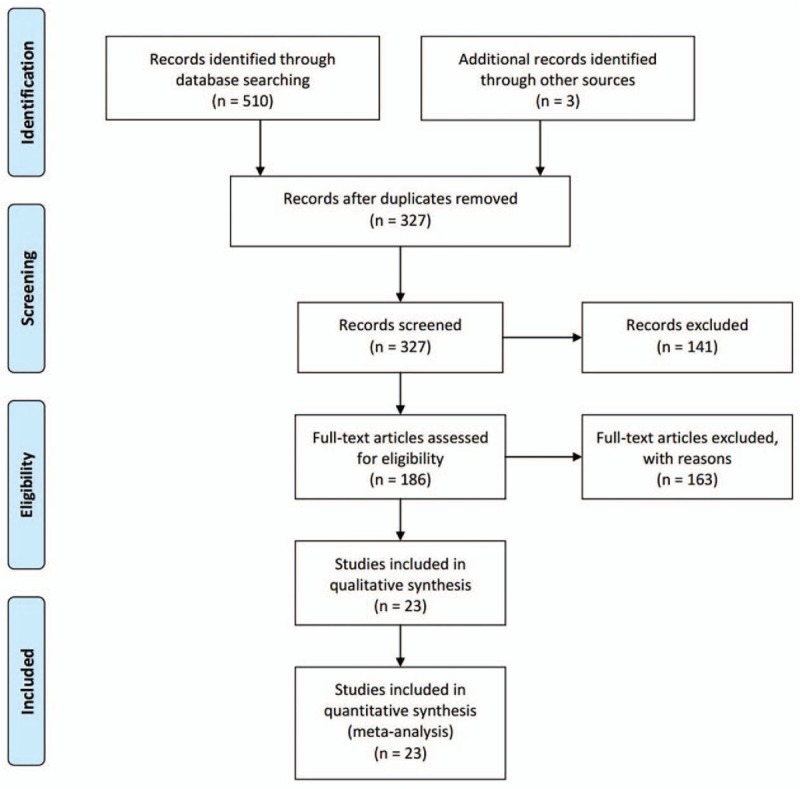
Preferred Reporting Items for Systematic Reviews and Meta-Analysis (PRISMA) flowchart showing the study selection process.

The process of identifying eligible studies was summarized in Figure 1. A total of 510 citations were identified in the initial search. After removing duplicates (n = 186) and having found 3 articles by manually searching the journals, 327 potential articles remained to be evaluated for inclusion using full text. After examining the titles and abstracts, 186 potentially eligible studies were selected for full-text review. One hundred sixty-three articles were excluded for not meeting the selection criteria. Finally, 350,939 participants in 23 articles were included in the meta-analysis (Supplementary Table 2). Twenty-two articles contained PI (348,517 participants), 2 articles contained PA (42,874 participants), and none articles contained PP information. The articles were published between 1993 and 2012, and the size ranged from 2422 to 78,431 participants.

### 3.2 Overall prevalence

**Figure 2:**
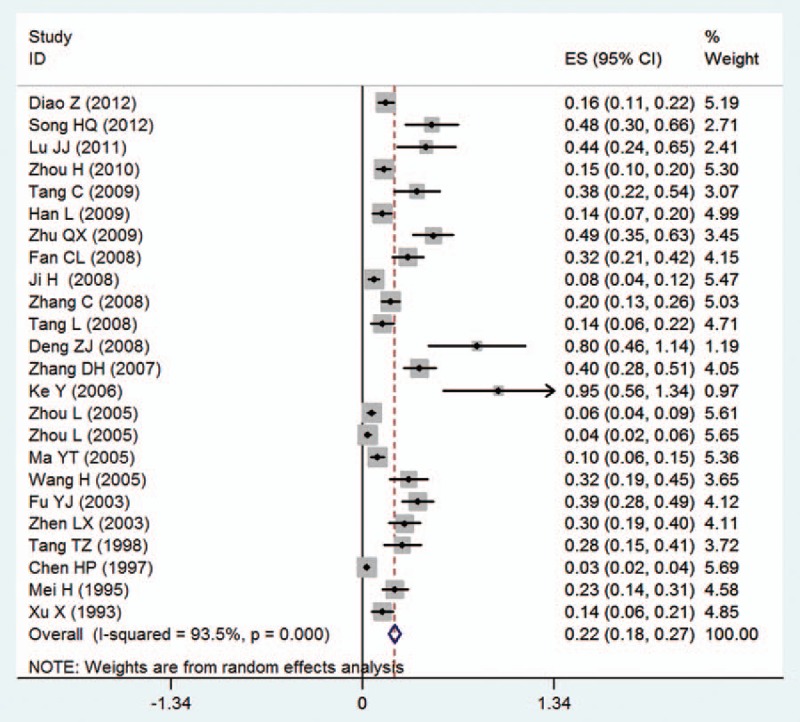
Forest plot of pooled estimated prevalence of abnormally invasive placenta in mainland China with corresponding 95% confidence intervals.

**Figure 3:**
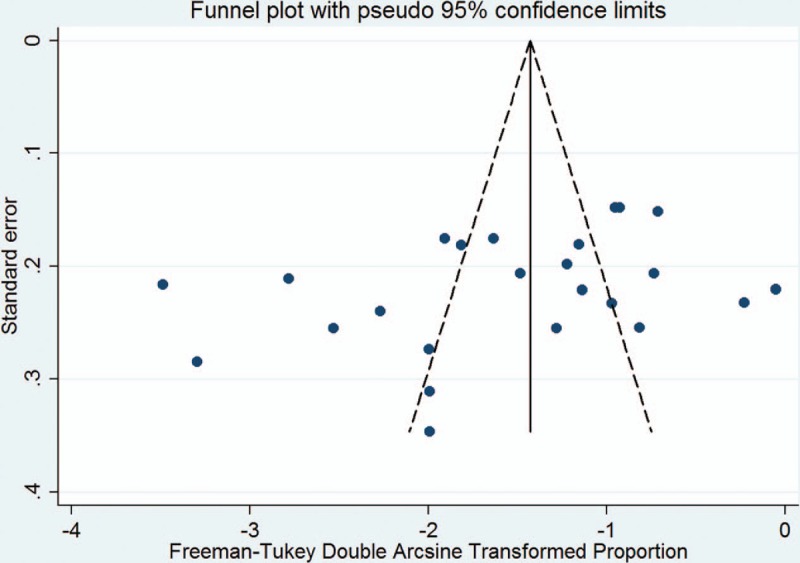
Funnel plot of the 23 studies included in the meta-analysis.

In this meta-analysis, the overall prevalence of AIP was 0.22% (95% CI 0.18%–0.27%) in a heterogeneous set of studies (*I*^2^ = 93.5%) (Fig. 2). In a subgroup of spectrums, the overall prevalence of PA and PI were 0.48% (0.39%–1.35%) with evidence of high heterogeneity (*I*^2^ = 95.1%) and 0.23% (0.18%–0.28%) with evidence of high heterogeneity (*I*^2^ = 93.7%), respectively. Although the funnel plot was slightly asymmetrical in overall prevalence (Fig. 3), both Begg test (*z* = −1.66, *P* = .097) and Egg test (*t* = −1.80, *P* = 0.085) showed no potential risk of publication bias.

### 3.3 Subgroup of PI

Because of only 2 articles in PA and none in PP, we could just be able to do further analysis in PI. We found 22 articles with >340,000 participants on the prevalence of PI. Table 1 summarized the subgroup pooled prevalence of PI by study characteristics such as geographical area (region and location), maternal age, survey year, quality score, and hospital level. Stratified analyses based on geographical area showed that the PI prevalence was similar in North (Beijing, Henan, Inner Mongolia, Shaanxi, and Tianjin) (0.23% [0.14%–0.32%]) and South (Guangdong and Sichuan) (0.23% [0.15%–0.32%]), and lower in Central (Anhui, Chongqing, Hubei, Hunan, Jiangsu, Shanghai, and Zhejiang) (0.20% [0.09%–0.31%]). The inlanders (Anhui, Beijing, Chongqing, Henan, Hunan, Inner Mongolia, Shaanxi, and Sichuan) (0.17% [0.12%–0.23%]) had a lower prevalence of PI than those living in coastal areas (Guangdong, Jiangsu, Shanghai, Sichuan, Tianjin, Zhejiang) (0.24% [0.35%–0.63%]). In maternal age groups, the prevalence of PI in age groups of <30 years and ≥30 years were 0.28% (0.20%–0.36%), and 0.16% (0.08%–0.23%), respectively. As time goes on, the prevalence of PI was higher and higher. Studies taking place in 2010 to present had a highest prevalence with a 0.48% (0.30%–0.66%), then 2000 to 2009 (0.28% [0.20%–0.35%]), 1990 to 1999 (0.20% [0.11%–0.29%]), 1980 to 1989 (0.14% [0.06%–0.21%]), and lowest with a 0.03% (0.02%–0.04%) in 1970 to 1979. The prevalence for the studies with lower quality score (0–5) was 0.21% (0.15%–0.26%), while for those in higher quality score (6–10), it increased to 0.28% (0.16%–0.39%). The tertiary hospital (0.21% [0.15%–0.26%]) had slightly lower PI prevalence than those in secondary hospital (0.26% [0.18%–0.33%]). Test for heterogeneity was significant in all the subgroups (Table 1).

**Table 1:**
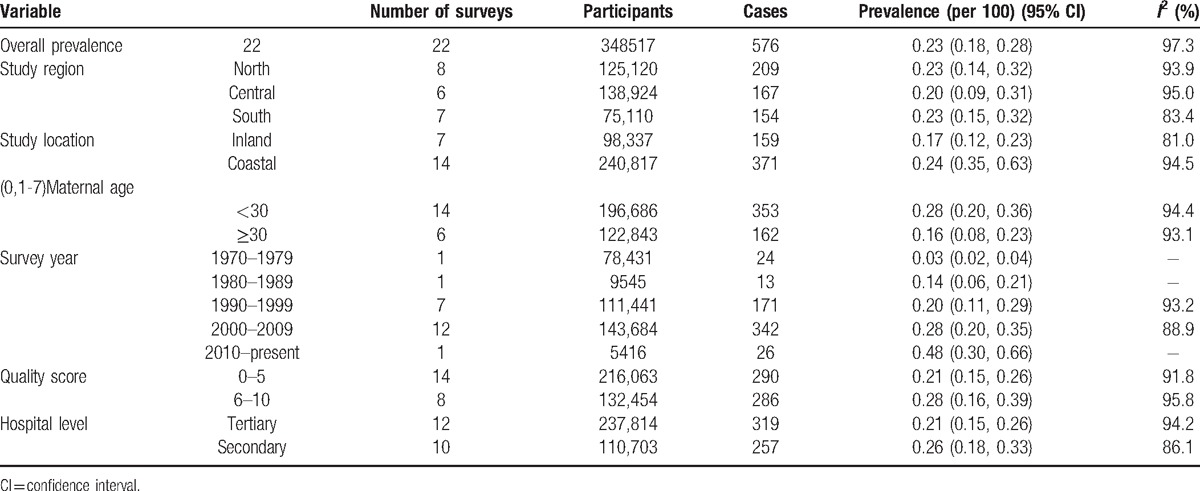
Prevalence of placenta increta in mainland China and subgroup analysis.

**Figure 4:**
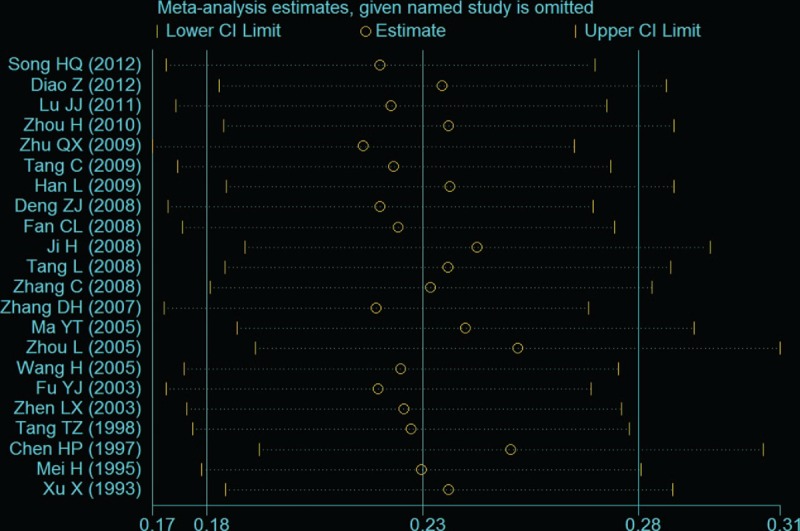
Sensitivity analysis for individual studies on the summary effect.

To explore the possible sources of heterogeneity, meta-regression was employed among the studies. Region, location, mean age, publication year, quality score, and hospital level, which may be potential sources of heterogeneity, were tested by meta-regression method. Through the univariate and age-adjusted regression model, none of aforementioned variables was significantly associated with the detected heterogeneity. However, the variable of publication year was significantly associated with heterogeneity in the stepwise multivariable analyses (*P* = .004) (Table 2). To confirm the stability and liability of the meta-analysis, sensitivity analysis was performed by calculating pooled PI prevalence again when any single study was deleted. Figure 4 showed that the corresponding pooled prevalence ranged from 0.22% (0.17%–0.26%) to 0.25% (0.19%–0.31%) and was not substantially altered. The statistically similar results indicated that each single study did not influence the stability of overall PI prevalence estimate in this meta-analysis.

**Table 2:**
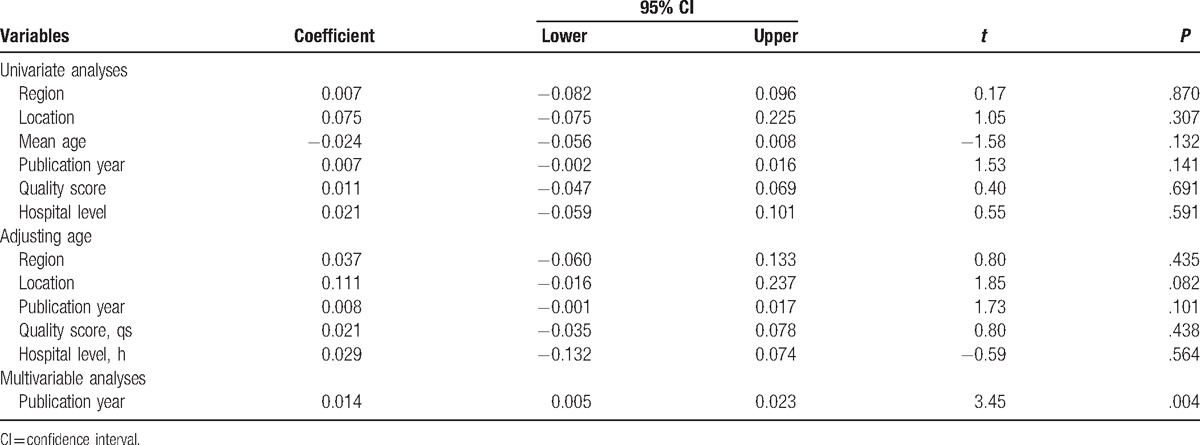
Metaregression analysis on the included studies in placenta increta.

## 4 Discussion

To our knowledge, this is the first systematic review and meta-analysis to investigate the prevalence of AIP among deliveries in mainland China. This meta-analysis based on 350,939 participants from 23 articles showed that overall AIP prevalence was 0.22%. In a subgroup, the prevalence of PA and PI was 0.48% and 0.23%, respectively. Although the funnel plot was slightly asymmetrical, both Begg and Egg test showed no potential risk of publication bias. There was substantial heterogeneity in the present meta-analysis, and we found the publication year was significantly associated with heterogeneity by the multivariable meta-regression. Most studies (22 articles) focused on the PI, only 2 articles on PA, and none on PP, and we cannot be able to further analysis in PA or PP. As the limitations noted above, the overall prevalence should be cautiously considered, especially in PA.

Via 2 routes, the interstitial and endovascular, extra villous trophoblast cell invading maternal decidua was vital to the maintenance of a successful pregnancy. In healthy pregnancy, maternal decidual defence barrier and placental angiogenesis were the perfect organizations to prevent the trophoblastic invasion. Excessive trophoblast invasion and decidual deficiency were the main mechanisms proposed for the pathophysiology of AIP.^[34]^ Multiparity, abortions, infections, uterine surgery or curettage, uterine irradiation, or endometrial ablation, which may cause decidual deficiency, were the potential risk factors, and, the scarring of the uterine wall was hypothesized as the most important risk factor leading to AIP.^[35]^ If the condition is not suspected beforehand, the main problem of severe AIP is the elevated maternal morbidity and even mortality owing to live-threatening bleeding at placenta delivery.^[14]^

The reported rate of AIP varies among different countries and even within the same country at different institutions. Still the frequency of severe AIP seems to have risen maybe 10 times during the last 50 years and approximately 1/500 to 1/2500 deliveries is complicated by AIP.^[14]^ American Society for Maternal-Fetal Medicine reported that the incidence has increased to 3 per 1000 deliveries in the last decade.^[4]^ In this meta-analysis study, we found that the overall prevalence of AIP was 0.22%, which was similar to the above report.

In the subgroup analysis of PI, we found the prevalence has increased significantly over time. This result is consistent with other studies, which attributed the cause to the increased rate of cesarean delivery. AIP can cause considerable maternal morbidity and mortality. In China, the primary-level hospital cannot provide effective treatment. They are asked to referral to higher-level hospital (secondary, or tertiary level). According to the Chinese hospital grade query system, we found that all included articles were from secondary- and tertiary-level hospital, and the prevalence was slightly higher in secondary-level hospital. In addition, our meta-analysis also revealed a tendency for the prevalence of PI to gradually decrease as the maternal age increased. This may be attributed to the uneven distribution of small sample size in different maternal age groups of this meta-analysis study.

In addition to the above reason, the following factors may also be considered. Since the Reform and Opening up in 1980s, sexual attitudes among Chinese people have changed considerably under the multiple influences of rapid modernization, urbanization, and exposure to Western culture.^[36]^ Unprotected premarital sex and unplanned pregnancy were increasingly accepted by young people in mainland China. Subsequently, abortions, which were devastating the endometrium, were also increasing over time. It has been reported that among unsafe abortions, approximately 15% involved girls aged 15 to 19 years and 26% involved young women aged 20 to 24 years.^[37]^

After enrollment expansion of colleges and universities, more and more young people get the chance to education. Age at first sexual intercourse was decreasing among these people. Although most believed that it was necessary to have knowledge about contraception and reproductive health, they still lacked it. The unplanned pregnancy rate among Chinese college students was approximately 34.03%.^[37]^ Meanwhile, unprecedented internal migration has been occurring in mainland China. Many young people have been moving to coastal and urban areas to seek better livelihood. Among female migrants, sexual and reproductive health problems such as unplanned pregnancies and induced abortions have constituted an important public health problem.^[38]^ The prevalence rate of induced abortion (41.6%), especially repeated abortions among migrant women, was much higher than that of the general population in China.^[39]^ This may also explain why the prevalence of PI in coastal was higher than inland.

A few policy implications can be drawn from this study to improve the prevalence of AIP. First, avoiding multiple cesarean deliveries or other uterine surgeries, without a specific indication, the obstetrician should strictly control the implementation of cesarean delivery in pregnant women, especially in primiparas. Second, government policy on reproductive health and contraceptive knowledge should be provided to high-risk group, such as, college students and migrant girls or women.

This meta-analysis had several limitations, which must be highlighted. There was high heterogeneity between studies, which was not explained by subgroup analyses and univariate andage-adjusted meta-regression. Therefore, the high heterogeneity was likely to be because of other study characteristics which were not measured or extracted in study population. Most articles focused on the PI, only 2 articles on PA, and none on PP, and we were not able to do further analysis in PA and PP. Moreover, the study samples of the included studies were based on hospital population, and there might have been some preferences while choosing and confounding, which could not be avoided. Nevertheless, the strength of the present meta-analysis lies in a large sample size (350,939 participants) from mainland China, and our study generated reasonably precise estimates of the prevalence of AIP.

## 5 Conclusions

To our knowledge, this is the first systematic review and meta-analysis to investigate the prevalence of AIP among deliveries in mainland China. The overall prevalence was 0.22%, and differed among different geographic areas and maternal age groups. As time goes on, the prevalence was higher and higher. The results would be useful for the design of AIP planning and implementation adequate health care systems and treatment programs in mainland China.

## Supplementary Material

Supplemental Digital Content

## Supplementary Material

Supplemental Digital Content
