# Supplementary material for: Prevalence of abnormally invasive placenta among deliveries in mainland China: A PRISMA-compliant Systematic Review and Meta-analysis
Source: Medicine (Baltimore). 2017 Apr 21;96(16):e6636. doi: 10.1097/MD.0000000000006636 (PMC5406078; doi:10.1097/MD.0000000000006636)
Supplement: Supplemental Digital Content [file medi-96-e6636-s002.doc]

Supplementary Table 2. Characteristics of the included studies and quality scores for assessing the risk of bias in the individual studies.

| Reference | First Author | Year | Cases | Participants | Age* | Period | Province | Geographical | Hospital Level | Outcome | Quality Score |
| --- | --- | --- | --- | --- | --- | --- | --- | --- | --- | --- | --- |
| 1 | Diao Z | 2012 | 33 | 20237 | 19 - 40 | 200501-201101 | Zhejiang | Ce/Co | Tertiary | PI | 6 |
| 2 | Song HQ | 2012 | 26 | 5416 | - | 201009-201207 | Henan | N/I | Secondary | PI | 4 |
| 3 | Lu JJ | 2011 | 18 | 4062 | 20 - 42 | 200706-200912 | Inner Mongolia | N/I | Tertiary | PI | 5 |
| 4 | Zhou H | 2010 | 35 | 23572 | 25 - 41 | 200501-201001 | Sichuan | S/I | Secondary | PI | 5 |
| 5 | Zhu QX | 2009 | 46 | 9363 | 25.54 - 2.41 | 199806-200103 | Mixed 3 Province | - | Tertiary | PI | 9 |
| 6 | Tang C | 2009 | 21 | 5536 | 21 - 38 | 200501-200801 | Sichuan | S/I | Secondary | PI | 5 |
| 7 | Han L | 2009 | 16 | 11765 | 21 - 32 | 199010-200608 | Shaanxi | N/I | Secondary | PI | 4 |
| 8 | Zhang C | 2008 | 35 | 17918 | 22 - 39 | 199705-200705 | Beijing | N/I | Tertiary | PI | 9 |
| 9 | Fan CL | 2008 | 33 | 10460 | - | 200101-200712 | Shaanxi | N/I | Secondary | PI | 6 |
| 10 | Tang L | 2008 | 11 | 8060 | 20 - 43 | 200201-200801 | Guangdong | S/Co | Secondary | PI | 5 |
| 11 | Ji H | 2008 | 18 | 22597 | 27.79 - 5.11 | 200101-200612 | Tianjin | N/Co | Tertiary | PI | 5 |
| 12 | Deng ZJ | 2008 | 21 | 2633 | 18 - 42 | 199501-200501 | Sichuan | S/I | Secondary | PI | 4 |
| 13 | Zhang DH | 2007 | 48 | 12094 | 21 - 41 | 200301-200610 | Hunan | Ce/I | Tertiary | PI | 6 |
| 14 | Ke Y | 2006 | 23 | 2422 | 31 - 6 | 200308-200503 | Shanghai | Ce/Co | Secondary | PA | 8 |
| 15.1 | Zhou L | 2005 | 15 | 40452 | 32 - 4 | 199401-200312 | Beijing | N/I | Tertiary | PI | 6 |
| 15.2 | Zhou L | 2005 | 25 | 40452 | 32 - 4 | 199401-200312 | Beijing | N/I | Tertiary | PA | 6 |
| 16 | Ma YT | 2005 | 20 | 19346 | 22 - 39 | 199201-200501 | Guangdong | S/Co | Secondary | PI | 5 |
| 17 | Wang H | 2005 | 23 | 7152 | 22 - 40 | 199801-200312 | Chongqing | Ce/I | Tertiary | PI | 5 |
| 18 | Fu YJ | 2003 | 48 | 12450 | 20 - 38 | 198707-200207 | Henan | N/I | Secondary | PI | 6 |
| 19 | Zhen LX | 2003 | 28 | 9480 | 26 - 40 | 199610-200204 | Guangdong | S/Co | Tertiary | PI | 6 |
| 20 | Tang TZ | 1998 | 18 | 6483 | - | 198301-199701 | Sichuan | S/I | Tertiary | PI | 4 |
| 21 | Chen HP | 1997 | 24 | 78431 | 22 - 45 | 196001-199501 | Hubei | Ce/I | Tertiary | PI | 5 |
| 22 | Mei H | 1995 | 26 | 11465 | 26 ± 3.23 | 199001-199406 | Jiangsu | Ce/Co | Secondary | PI | 5 |
| 23 | Xu X | 1993 | 13 | 9545 | 21 - 41 | 198501-199101 | Anhui | Ce/I | Tertiary | PI | 5 |

*Age was shown in mean ± standard deviation or minimum – maximum.

PA, placenta accreta; PI, placenta increta.

N: north; Ce: central; S: south. I: inland; Co coastal.

**References**

1. Diao Z, Chen XJ, Chen YQ, Zhang WM, Huang YP. Clinical analysis of 33 postpartum patients with placenta implantation. Maternal and Child Health Care of China. 2012;27(14):2109-11.

2. Song HQ. Treatment of Placenta Increta in 26 Cases. Chinese Community Doctors. 2012;14(34):150.

3. Lu JJ, Qi MG. The Clinical Analysis of 18 Cases of Placenta Increta. Journal Of Inner Mongolia Medical College. 2011;33(3):239-41.

4. Zhou H, Li Y. Clinical analysis of the placenta accreta. Sichuan Med J. 2010;31(12):1764.

5. Zhu QX, Gao ES, Chen AM, Luo L, Cheng YM, Yuan W. Mifepristone-induced abortion and placental complications in subsequent pregnancy. Human reproduction (Oxford, England). 2009;24(2):315-9. Epub 2008/12/05. doi: 10.1093/humrep/den426. PubMed PMID: 19054774.

6. Tang C, Li DQ. The Clinical Analysis of 21 Cases of Placenta Increta. Chinese Community Doctors. 2009;11(216):65-6.

7. Han L, He YL. The Clinical Analysis of 16 Cases of Placenta Increta. Med Forum. 2009;13:700-01.

8. Zhang C, Liu XY, Fan GS, Yang JQ, Liu JT, Bian XM. Clinical analysis of 47 cases of placenta accreta in the second and third trimesters. Chin J Obstet Gynecol. 2008;43(7):506-9. Epub 2008/12/17. PubMed PMID: 19080513.

9. Fan CL. Diagnosis and Treatment of 33 Cases with Implanted Placenta. JPMT. 2008;15(25):3391.

10. Tang L, Li X. The Clinical Analysis of 11 Cases of Placenta Increta. J Gannan Med Uni. 2008;28(4):538-39.

11. Ji H. The Clinical Analysis of 17 Cases of Placenta Increta. Tianjin Med J. 2008;36(8):646-47.

12. Deng ZJ. The Clinical Analysis of 26 Second trimester pregancies of Placenta Increta. Ningxia Med J. 2008;30(1):72-3.

13. Zhang DH, Jin MH. The Clinical Analysis of 48 Cases of Placenta Increta. J Clin Res. 2007;24(5):853-55.

14. Ke Y, Lu JH, Yang BL, Guo HQ, Ma QY, Zhu H, et al. Involvement of matrix metalloproteinase-2, -9, and tissue inhibitors of metalloproteinase-1, 2 in occurrence of the accrete placenta. Chin J Obstet Gynecol. 2006;41(5):311-4. Epub 2006/06/10. PubMed PMID: 16762185.

15. Zhou L, Wu LF. Retrospective Analysis of 40 Cases of Placenta Increta. J Capital Uni Med Sci. 2005;26(1):90-2.

16. Ma YT, Ling Y, Liu MX. The Clinical Analysis of 20 Cases of Placenta Increta. JPMT. 2005;12(8):2154-55.

17. Wang H, Zhang JH, Wu WX. The Clinical Analysis of 23 Cases of Placenta Increta. J Prac Obste & Gyne. 2005;21(2):93.

18. Fu YJ, Wang SM, Wang YR. Clinical Analysis of Cases of Placenta Accreta. Chinese Journal for Clinicians. 2003;31(7):35-6.

19. Zheng LX, Li SJ, Chen DX. The risk factors of placenta increta and treatment in 28 cases. Chinese Journal of Practical Gynecology and Obstetrics. 2003;19(4):239-40.

20. Tang YZ. Clinical Analysis of 18 Cases of Placenta Accreta. Journal of North Sichuan Medical College. 1998;13(3):52-3.

21. Chen HP, Xu JP, Lu QY, Ma TY. The Clinical Analysis of 24 Cases of Placenta Increta. Acta Medicinae Universitatis Scientiae et Technologiae Huazhong. 1997;26(3):241.

22. Mei H. The Clinical Analysis of 26 Cases of Placenta Increta. J Prac Obste & Gyne. 1995;11(6):314-15.

23. Xu X. The Clinical Analysis of 13 Cases of Placenta Increta. Acta Universitatis Medicinalis Anhui. 1993;28(3):224.
